# An Educational Need Regarding Treatment-Related Infertility and Fertility Preservation: a National Survey Among Members of the Dutch Society for Medical Oncologists

**DOI:** 10.1007/s13187-021-02084-1

**Published:** 2021-10-08

**Authors:** E. M. Krouwel, E. M. L. Birkhoff, M. P. J. Nicolai, S. Osanto, H. Putter, R. C. M. Pelger, H. W. Elzevier

**Affiliations:** 1 Department of Urology, Leiden University Medical Centre, Leiden, the Netherlands; 2 Department of Medical Decision Making, Leiden University Medical Centre, Leiden, the Netherlands; 3 Department of Urology, Netherlands Cancer Institute-Antoni van Leeuwenhoek Hospital, 1066 CX Amsterdam, The Netherlands; 4 Department of Oncology, Leiden University Medical Centre, Leiden, the Netherlands; 5 Department of Medical Statistics, Leiden University Medical Centre, Leiden, the Netherlands

**Keywords:** Cancer, Fertility, Cryopreservation, Fertility preservation, Oncologist, Quality of life

## Abstract

Cancer diagnosis and treatment may influence reproductive planning and impact fertility in patients of reproductive age. Although guidelines have been established in the past decade, education, practice, and attitudes of medical oncologists regarding fertility preservation remain undecided. A nationwide survey was performed among members of the Dutch Society for Medical Oncology. Demographics, practice, knowledge, and barriers were measured regarding information provision of fertility preservation towards cancer patients of childbearing age. From 392 members, 120 oncologists completed the questionnaire (30.6%). Majority of oncologists was convinced it is their responsibility to discuss impact of cancer treatment to fertility (93.2%), yet 68.3% discussed the subject often or always (*n* = 82). Oncologists employed in district general hospitals were less likely to discuss fertility (*p* = 0.033). On average, 44.6% of reproductive men and 28.9% of reproductive women is referred to fertility specialists. Half of the respondents declared to possess sufficient knowledge regarding fertility preservation (*n* = 57, 47.5%). Poor prognosis (53%), unlikely survival (43.1%), and high chances on fertility recovery (28.7%) were identified as barriers to discussing fertility preservation. Among oncologists, impact of cancer treatment on fertility is a well-accepted responsibility to counsel. Despite, self-reported knowledge regarding fertility preservation is strongly varying. In practice, fertility is discussed to some extent, influenced by several barriers and depending on prognosis and type of hospital. Patients benefit from knowledge improvement among oncology care providers concerning fertility effects of cancer treatment. Education during medical school, residency, and among practicing oncologists may raise awareness, together with enhancement of referral possibilities.

**Supplementary Information:**

The online version contains supplementary material available at 10.1007/s13187-021-02084-1.

## Introduction

Cancer treatments are associated with a variety of undesirable side effects; of which one of specific concern to young men and women is the effect on their endocrine health and future reproductive ability [38]. Due to the increasing 5-year, and overall survival over the past few decades, consideration for physical and psychological consequences becomes progressively prioritized [11]. Loss of fertility is a devastating side effect for young cancer survivors with severe emotional impact [2], and specifically resulting from treatment with chemotherapy or radiation therapy [16, 27, 32]. Moreover, the prospect of facing treatment-induced infertility for women of reproductive age is proven to affect their cancer treatment decisions in up to 41% [22, 34].

Cytostatic cancer drugs are designed to target dividing cells, implying that in addition to inhibiting cancer cell growth, proliferating primordial follicles which enfold oocytes are conjointly harmed [6]. As for men, infertility and persistent azoospermia is a common long-term adverse effect [7]. Alkylating drugs are feared most for their effect to fertility, by inducing both impaired fertility and early menopause [33]. Although the exact risk for cancer- and treatment-related infertility depends on the chemotherapy agent, the chemotherapeutic regime, and the age of the patient, it should not be underestimated considering the long-term impact. Being the physician prescribing cytostatic drugs, the medical oncologist is responsible for informing about the risk of infertility before commencing a gonad toxic treatment, referral to a reproductive specialist for fertility preservation (FP) and discussing alternative treatment strategies if applicable. Nonetheless, various studies suggest the information provision regarding fertility issues is often experienced as inadequate by patients. Furthermore, it is suggested that cancer care physicians do not possess sufficient knowledge regarding fertility risks and options for FP [8, 13, 25, 36]. As a result, information is not timely provided or in some cases is not provided at all [3, 4, 23]. At the time of diagnosis, fertility issues are often outweighed by the focus on survival. A Dutch observational study, showed in 2011, a total of 9.8% of female patients were referred for FP counselling. However, the absolute numbers of patients receiving FP counselling increased over time [5]. And indeed, informing cancer patients of reproductive age about possible reduced fertility and referral to a reproductive specialist in a timely manner is recommended by national [18, 29], European [31], and international guidelines [24]. Fertility counselling performed by a fertility specialist prior to cancer treatment, in comparison to the oncologist alone, is associated with better psychological health. Those patients who undertook counselling and proceeded with FP reported reduced regret, compared with those who did not proceed to FP [22]. Patients who felt fertility concerns have not been given full consideration at the time of diagnosis have been shown to cope with psychological distress, expressed by uncertainty and concern, as well as higher levels of depression and cancer or fertility‐related trauma during survivorship [22].

Over the last decade, we have experienced a surge of scientific reports on aspects of altered fertility in young adults with cancer; these particularly include the growing number of available preservation options [1] and the devastating impact of the loss of fertility to cancer survivors [9, 30, 37]. In addition, various studies investigated practice regarding (referral for) FP counselling by physicians involved with oncology patients. An overview of quantitative studies among oncology care providers published in the past 10 years regarding knowledge level, the discussion of fertility, referral to fertility specialists, and barriers regarding this discussion is provided in Supplement 1. Paediatric studies have been excluded in this overview.

To date, published quantitative surveys have suggested there may be a deficiency in medical oncologists’ knowledge about FP options and that the provision of information to patients about FP may be suboptimal. The purpose of the hereby presented nationwide study was to evaluate Dutch medical oncologists’ practice patterns, knowledge, educational need, attitudes, and barriers regarding treatment-related infertility and FP among men and women of reproductive age.

## Methods

### Study Design and Cohort Identification

A questionnaire was used for collecting data in a cross-sectional postal survey. The sample consisted of 433 members of the NVMO (Dutch Society for Medical Oncology) with several areas of expertise. Our sampling strategy aimed for representation with regard to different tumours, employment setting, level of education, years of oncology experience, type of hospital, age, and gender.

### Instrument Design and Development

The questionnaire was developed by the authors. The content of the questionnaire was evaluated by 4 oncologists working in Leiden University Medical Center through an anonymous pilot study and modified using their feedback. The final version comprised a demographic sheet, including professional background, experience in oncology practice, gender, and age. Furthermore, Likert-scale items measured practices, attitudes, content of sexual and fertility counselling, responsibility, need for education, and barriers regarding the discussing of sexual function and fertility issues. In addition, a list was made of most common medications measuring knowledge about their possible side effects to sexual function, to future reproductive ability and regarding teratogenicity. Data concerning the discussing of sexuality issues and knowledge about medication were processed separately [19].

### Survey Administration

The questionnaires were sent to all medical oncologists who were member of the NVMO; members not practicing in the Netherlands have been excluded. After the initial mailing was finished, reminders were sent to non-responders after 6 and 12 months. An information letter concerning the study and a post-paid return envelope were added, as well as an opt-out possibility. Data were collected anonymously in order to prevent a self-reporting bias.

### Analysis

Data analysis was performed using SPSS (Release 22; SPSS Inc., USA). Frequency distribution was used to calculate demographic information. Respondents were recoded regarding age (set at median age 47: 46 years or under vs. over 46), experience (0–10 years vs. 11 years or more), knowledge (none to some vs. sufficient to a lot), and residents vs. qualified specialists. Observed differences between demographic information and specific answers were identified using the Pearson’s chi-square test, linear-by-linear association, paired *T*-test, and independent sample *T*-test. *P-*values < 0.05 were considered statistically significant.

## Ethics

In the Netherlands, studies that do not involve patients or interventions are not subject to approval from an ethical board. The ethical board was consulted for a comparable previous study, as the study did not concern information recorded by the investigator in such a manner that subjects could be identified, and as it did not compromise the study participants’ integrity, the Committee declared that no official ethical approval was needed.

## Results

### Demographics

Of the 433 invited participants, 209 responses were received, resulting in a response-rate of 48.3%. Of the 209 responses, 26 oncologists reported to be retired, 6 physicians were not medical oncologists, and 9 were returned to sender. Forty-eight oncologists reported they were not willing to participate due to a lack of time (*n* = 35), a lack of interest (*n* = 2), a lack of experience (*n* = 2), the length of the instrument (*n* = 4), or other reasons (*n* = 5). Out of 392 eligible participants, 120 oncologists completed the questionnaire (30.6%). Majority of the respondents reported breast cancer (73.3%) and colorectal cancer (65.8%) as area of expertise (multiple areas of expertise possible). For extensive information on the respondents’ characteristics, see Table 1.

**Table 1:** Demographic characteristics

Demographic characteristics (*n* = 120)	*n* (%)
Age (years)	
Mean 45.8	119 (99.2)
Median 47 (range 30–64)	
Gender	
Male	56 (46.7)
Female	63 (52.5)
Unknown	1 (0.8)
Oncology experience (years)	
1–2	19 (15.8)
3–5	27 (22.5)
6–10	13 (10.8)
11–15	19 (15.8)
> 15	40 (33.3)
Unknown	2 (1.7)
Function	
Oncologist	74 (61.7)
Oncologist and haematologist	15 (12.5)
Haematologist	12 (10)
Resident medical oncology	19 (15.8)
Hospital type	
University hospital	40 (33.3)
Top clinical teaching hospital	27 (22.5)
District general hospital	47 (39.2)
Categorical cancer hospital	3 (2.5)
University hospital and district general hospital	2 (1.7)
Unknown	1 (0.8)
Areas of interest^a^	
Breast cancer	88 (73.3)
Colorectal cancer	79 (65.8)
Palliative medicine	57 (47.5)
Urological cancer	55 (45.8)
Gynaecological cancer	53 (44.2)
Haematology	37 (30.8)
Lymphoma	32 (26.7)
Neuroendocrine cancer	15 (12.5)
Head and neck cancer	14 (11.7)
Melanoma	9 (7.5)
Sarcomas	8 (6.7)
Lung cancer	3 (2.5)
Experimental research (phase I-II)	3 (2.5)
Gastro-intestinal	2 (1.7)
Hepatic and biliary tract cancer	1 (0.8)

Multiple areas of interest possible

### Practice

When it comes to discussing the impact of cancer treatment to fertility, almost seventy percent of respondents (*n* = 82, 68.3%) stated to discuss fertility often or always, 20% (*n* = 24) declared to discuss it in more than half of the cases, 5.8% (*n* = 7) in half of the cases, 3.3% (*n* = 4) in less than half of the cases, and 2.5% (*n* = 3) hardly never/never. Among oncologists working in a district general hospital, it was less usual to discuss fertility. In district general hospitals, 84.1% discussed fertility in more than half of the cases vs. 90.3% in all others hospitals (*p* = 0.033, linear-by-linear). No significant differences were observed regarding gender, all different specialties, residents vs. oncologists, age through 46 years or older, experience through 10 years or more, level of knowledge, and availability of a department protocol.

When cancer treatment has the prospect to cure, almost all respondents (*n* = 114, 98.3%) discuss fertility. Yet, if cancer treatment has no prospect of cure, only half of the respondents (*n* = 61, 52.6%) discuss fertility. When treatment is at palliative stage, a quarter of the respondents stated to discuss fertility (23.3%, *n* = 27).

Fertility is discussed with women until the age of 44 on average (range 35–100 year, SD 8.2, *n* = 114) and on average with men until the age of 53 (range 37–100, SD 10.6, *n* = 107).

Topics that were reported to be discussed with women are a desire to start a family (*n* = 120, 100%), menopausal symptoms (*n* = 105, 87.5%), fear for premature termination of pregnancy (*n* = 15, 12.5%), fear for congenital abnormalities (*n* = 51, 42.5%), and heredity (*n* = 79, 65.8%). With men, frequently discussed topics were a desire to start a family (*n* = 117, 97.5%), erectile dysfunction (*n* = 60, 50%), ability to ejaculate (*n* = 24, 20%), fear for congenital abnormalities (*n* = 39, 32.5%), and heredity (*n* = 63, 52.5%).

### Knowledge

To the question: ‘How much knowledge do you possess regarding FP for cancer patients?’, 47.5% of the respondents (*n* = 57) answered sufficient knowledge, 41.7% (*n* = 50) had some knowledge, 7.5% (*n* = 9) said they did not have much knowledge, and 3.3% (*n* = 4) reported they possessed a lot of knowledge. Oncologists estimated their knowledge significantly higher in comparison to residents (linear-by-linear association *p* = 0.004).

Three-quarters of the oncologists (*n* = 86, 75.4%) would like to improve their knowledge towards fertility issues and management of fertility issues. Residents significantly more often wish to improve their knowledge (*p* = 0.041). Experience, however, is not of significant influence to the wish for more knowledge (*p* = 0.081). Almost three-quarters (*n* = 84, 74.3%) of the respondents believe there is too little attention for fertility issues and management of fertility issues during residency. Respondents estimated that initial cryopreservation of semen would cost 693,15 euro (range 30–15,000 euro; SD 1801; *n* = 75) with an annual cost for cryopreservation of 103,07 euro (range 0–500 euro; SD 124.8; *n* = 75).

### Local Practice

Approximately 40% (*n* = 45, 38.5%) of the respondents reported a protocol or a standard at their current workplace stating the routine discussing of fertility, 41.9% (*n* = 49) did not have such a protocol, and 19.7% (*n* = 23) was not aware of such a protocol. During multidisciplinary oncology meetings, according to 37.1% (*n* = 43), fertility is regularly discussed. Seventy-three oncologists (62.9%) reported fertility is not routinely discussed in multidisciplinary meetings. Half of the oncologists reported there is sufficient patient information available in their department regarding fertility (*n* = 56, 48.3%), a quarter (25%, *n* = 29) reported there is not sufficient information, and the remaining quarter (26.7%, *n* = 31) did not know whether patient information is available. Seventy percent often or always registered fertility consultation in the patient’s file (*n* = 82), 12% (*n* = 14) does so in more than half of the cases, and 21 oncologists (18%) reported to register in half of the cases or less. Eighty-four percent (*n* = 101) never/hardly never prescribed a gonadotropin-releasing hormone agonist (GnRH–A) before starting chemotherapy, for protection of the ovaria. Twelve oncologists (10%) did so in less than half of the cases, 2.5% (*n* = 3) in half of the cases, and 3.3% (*n* = 4) did so in more than half of the cases or almost always.

### Responsibility

According to 36.4% (*n* = 43), responsibility for the discussion of fertility was assigned to their department, 53 oncologists (44.9%) stated there were no agreements regarding responsibility and 18.6% (*n* = 22) did not know if there were agreements. Majority of the participants 93.2% (*n* = 110, question answered by *n* = 118) was convinced that it is the responsibility of the oncologist to discuss fertility with patients of reproductive age; 5.8% (*n* = 7) disagreed to this responsibility. One oncologist did not know whether it is within the treatment responsibility to discuss fertility. In addition, 78% (*n* = 92) believed that there is also a responsibility for the oncology nurse, 17% (*n* = 20) did not believe that it is the oncology nurses’ responsibility to address fertility, and 5.1% (*n* = 6) did not know if it should be oncology nurses’ responsibility.

### Barriers

The respondents were given a list of 30 possible barriers for discussing fertility, in order for them to indicate to which extent they agreed. The three barriers most agreed upon by the respondents were ‘prognosis is poor’ (53%), ‘unlikely patient will survive treatment’ (43.1%), and ‘high chance on fertility recovery after treatment’ (28.7%). The three barriers most disagreed upon by respondents were ‘patient cannot afford treatment’ (91.3%), ‘patient is single’ (90.6%), and ‘no contact information available of fertility specialist’ (88.8%). A complete overview of barriers can be found in Table 2.

**Table 2:** Barriers towards discussing fertility

I would tend not to discuss fertility with a patient because:	Agree	Neither agree nor disagree	Disagree
	*n* (%)	*n* (%)	*n* (%)
Patient cannot afford treatment	1 (0.9)	9 (7.8)	105 (91.3)
Patient is single	2 (1.7)	9 (7.7)	106 (90.6)
No contact information available of fertility specialist	2 (1.7)	11 (9.5)	103 (88.8)
Patient is a teenager	3 (2.8)	4 (3.7)	101 (93.5)
This may raise fear and discomfort	3 (2.5)	20 (17)	95 (80.5)
Semen cryopreservation is not adolescent friendly	3 (2.7)	20 (17.7)	90 (79.6)
Uncomfortable to discuss fertility	4 (3.5)	23 (20.4)	86 (76.2)
Patient already has a child/children	5 (4.3)	22 (18.8)	90 (76.9)
Semen cryopreservation is expensive for patient	5 (4.5)	28 (25.2)	78 (70.3)
Hereditary tumour (risk of passing to child)	7 (6)	17 (14.5)	93 (79.5)
Pregnancy during or after chemotherapy may induce malformation of child	8 (6.9)	26 (22.4)	82 (70.7)
I do not possess enough knowledge regarding fertility preservation options	8 (6.8)	27 (23.1)	82 (70.1)
Patient is homosexual	10 (9)	19 (17.1)	82 (73.9)
Fear of possible malignant transformation of ovarian tissue	11 (9.7)	18 (15.9)	84 (74.3)
Fertility treatment may influence success of cancer treatment	12 (10.3)	27 (23)	79 (66.7)
Treatment delay	12 (10.3)	27 (23.1)	78 (66.7)
Patient does not bring up the subject	13 (11)	25 (21.2)	80 (67.8)
Possibility of reintroducing cancer or metastasis by ovarian tissue cryopreservation	13 (11.4)	26 (22.8)	75 (65.8)
Culture or religion of patient does not support assisted fertility	13 (11.5)	32 (28.3)	68 (60.2)
Curation has priority	19 (16.2)	33 (28.2)	65 (55.6)
Difficult to predict risk for deteriorated fertility	19 (16.4)	33 (28.4)	64 (55.2)
Ovarian tissue cryopreservation is experimental	20 (17)	36 (30.5)	62 (52.5)
Patient is treated before with chemo and/or radiotherapy	23 (19.8)	30 (25.9)	63 (54.3)
Lack of time during consult	25 (21.4)	22 (18.8)	70 (59.8)
Patient is HIV positive	28 (25.7)	26 (23.9)	55 (50.4)
Hormonal treatment may increase risk of recurrence	28 (25)	31 (27.7)	53 (47.3)
Patient is not able to ejaculate; therefore, cryopreservation is not possible	31 (27.4)	22 (19.5)	60 (53.1)
High chance on rapid recovery of reproductive function after treatment	33 (28.7)	31 (27)	51 (44.3)
Unlikely patient will survive treatment	50 (43.1)	25 (21.6)	41 (35.3)
Prognosis is poor	62 (53)	27 (23.1)	28 (23.9)

For ease of presentation, results in response categories ‘strongly agree’ and ‘agree’ have been merged, as have ‘strongly disagree’ and ‘disagree’. Total number of respondents may differ per barrier, as some respondents skipped barriers

### Referral to Fertility Specialist

When asked which percentage of all patients of reproductive age has been referred to a fertility specialist, on average 44.6% of men (range 0–100%; SD 37.1), and on average 28.9% of women (range 0–100%; SD 31.4), is referred. The percentage of women being referred is significantly lower in comparison to men (*p* < 0.001, paired *T*-test). The percentage of men being referred was more often by oncologists of 47 years and older (*p* = 0.028, ind. sample *T* test) and by residents (*p* = 0.001, ind. sample *T* test). There were no significant differences in gender or hospital type of the respondents in reference to the percentage of referred patients.

## Ethics

The participating oncologists were asked to give their opinion upon post-mortem use of preserved material for assistant reproduction for the partner. Half of the oncologists (*n* = 56) believed this is acceptable, 39 oncologists (35.5%) believed this is not acceptable, and 15 oncologists (13.6%) were not aware of the existence of this possibility.

## Discussion 

### Main Findings

Considering it is crucial that medical oncologists address the impact of cancer treatment to fertility with patients of childbearing age, this survey intended to represent current practice and knowledge among medical oncologists practicing in the Netherlands. Main findings of our study demonstrate an accountable attitude with regard to fertility issues among responding oncologists, yet about two-thirds of the participants stated to often or always discuss the impact to fertility. Practice behaviour is mainly influenced by patients’ prognosis, type of hospital, and fertility recovery chances. On average, 44.6% of reproductive men and 28.9% of reproductive women are referred to a fertility specialist. Half of our respondents said to possess sufficient knowledge concerning FP. Three-quarter of the oncologists believed too little training is paid to the subject during residency and expressed a wish for additional education on fertility issues and preservation options.

### Comparison to Literature and Interpretation of Findings

In the past decade, several international surveys have been performed amongst oncology care providers regarding the provision of FP (Supplement 1). Response-rates of previous surveys differ from 14 to 78.6% (mean 47.3%). Much of what is known about fertility and cancer is the result of studies conducted in the USA, the UK, and other countries. Two studies have been performed in the Netherlands, reflecting on the practice of physicians from several different cancer specialties, not solely medical oncologists. Our findings indicate that responsibility for fertility concerns is acknowledged by oncologists; however, in practice, the discussing of fertility concerns may vary. Other surveys among oncologists across the world show greatly varying results, with discussing percentages ranging from 13.6 to 98% and referral percentages from 15 to 97% (Supplement 1). Although the counselling percentages vary in countries and regions, it is clear that we are facing a generic concern probably applicable to all cancer care institutes in a greater or lesser extent.

In comparison to previous surveys worldwide, the surveyed oncologists score average on discussing fertility concerns in practice. As for referral to a fertility specialist, scores of the Dutch oncologists are slightly lower than average. However, most of the reviewed surveys were conducted in single centres, selected populations (e.g. among oncologists who had previously enrolled women on premenopausal studies) or demarcated areas, and often only investigated FP regarding female cancer or breast cancer patients as seen in Supplement 1. Therefore, the questioned populations may be biased, as local practices may differ significantly. Differences in several types of practices are empowered by our finding that oncologists employed in a district general hospital were less likely to discuss fertility issues. This proven variety in practice between district general hospitals and top clinical and university hospitals may be explained by limited access to fertility departments which are usually located in university or top clinical teaching hospitals. This phenomenon was previously observed by Hariton et al., showing the association between the establishment of a oncofertility clinic and increased consultations for FP [17]. In addition, a recent Dutch survey showed the relevance of available reproductive specialists with specific expertise with regards to women with cancer, as a lack of available specialists was reported a major barrier against discussing fertility issues with patients [25]. Furthermore, Louwe et al. revealed a positive correlation between the number of FP options available and the number of information sources available in regard to confidence in the physicians’ knowledge [25]. Negative correlation was shown between the frequency of discussion fertility issues and a lack of reproductive specialists in the geographic region, which is very similar to our results.

Barriers most mentioned by our respondents were a poor prognosis and unlikelihood the patient will survive the treatment. In comparison to literature, these are often mentioned barriers towards discussing fertility issues by clinicians working in oncology departments. By way of comparison, in a Swedish survey, the barrier ‘poor prognosis’ was mentioned by 78% [28], in a German survey by 62.7% [8], in a Dutch pilot survey by 62% [26], in a Canadian survey by 66.4% [39], in a UK survey by 78.6% [15], and in an American survey by 30% [14]. Besides medical reasons, one out of five oncologists stated lack of time during the consult as a barrier towards discussing fertility.

In addition, we asked the clinicians to estimate costs of semen cryopreservation. Estimations of the costs of semen cryopreservation were variable; on average, the estimated costs were fairly overestimated. Specifying, costs for initial cryopreservation were estimated €693,15, actual costs are €119,82, with additional costs per sample of €62,81 (reimbursements 2018). Annual costs for cryopreservation were estimated €103,07, actual annual costs are €60,12 (reimbursements 2018). As the costs are fairly overestimated (specifically the initial costs), patients may be informed incorrectly by their clinicians. In some cases, this may result in the decision to withhold from cryopreservation, an undesirable consequence.

Clearly, there is a reported lack of training for fertility issues and their management during residency. Consequently, a wish for additional education is expressed by the majority of the respondents, implying a major role for the development of training courses and implementation of the subject fertility issues during residency. By these means, early referral by oncologists before initiation of chemotherapy and radiotherapy will be enabled, as this is a key factor for success in (female) FP [35].

### Strengths and Limitations

The completion rate of 30.6% is lower than the average response rate of physicians surveys [10], also on the lower limits in comparison to physicians surveys performed in the Netherlands with response rates running from 28 to 55% [12, 20, 21]. The completion rate may be explained by the length of the questionnaire, the content of the questionnaire (assessing treatment side-effect knowledge, which may be embarrassing if unfamiliar with this knowledge), and the sensitivity of the topics sexuality and fertility. Yet, a non-response bias may have occurred. Oncologists with affinity for the subject may have been more inclined to answer than oncologists who are less committed to fertility concerns. Demographic characteristics of non-responders have not been made available; consequently, non-response calculations could not be made. A non-validated questionnaire has been used as a validated instrument was not available. Nevertheless, a pilot study has been conducted to check for validity and reliability. Subdivision by area of specialization resulted in small numbers of medical oncologists in each specialization group. Accordingly, it was not possible to do sub analyses for every separate area of specialization.

### Clinical Implications

Awareness and sufficient knowledge among medical oncologists regarding possible toxic effects to endocrine and reproductive health is of critical importance for young men and women with cancer. Due to a lack of knowledge, referral possibilities, and counselling barriers, the ability to start or complete a family after treatment may not be extensively discussed. Subsequently, many men and women of reproductive age with cancer could be missing the opportunity to investigate their FP options. We recommend expansion of education of fertility treatment risks and preservation options starting in medical school, continued during residency training and updates when practicing as a medical oncologist. A culture of shared decision making should be pursued, through the development of clear fertility referral pathways including psychosocial support to improve care for men and women of childbearing age facing a cancer treatment.

## Conclusion

In conclusion, the results suggest that medical oncologists take responsible attitudes towards fertility preservation in oncology practice. Self-reported knowledge regarding fertility preservation is strongly varying and the majority expressed a wish for additional education. Practice attitudes remain under influence of factors like poor prognosis, a lack of knowledge, treatment-delay, and local availability of fertility specialists. Efforts to develop educational training on treatment fertility risks, communication skills, and acquaintance with fertility preservation options are highly recommended. Improvement of awareness regarding fertility preservation and in addition availability of fertility specialists in district general hospitals may increase referral of young cancer patients for fertility preservation. Timely referral to discuss preserving options for endocrine and reproductive health is crucial, before irreversible damage to the gonads is done.

## Supplementary Information

Below is the link to the electronic supplementary material.

Supplementary file1 (DOCX 46 KB)
