# Supplementary material for: An Educational Need Regarding Treatment-Related Infertility and Fertility Preservation: a National Survey Among Members of the Dutch Society for Medical Oncologists
Source: J Cancer Educ. 2021 Oct 8;38(1):106–14. doi: 10.1007/s13187-021-02084-1 (PMC9852179; doi:10.1007/s13187-021-02084-1)
Supplement: Supplementary file 1 — Supplementary file1 (DOCX 46 KB) [file 13187_2021_2084_MOESM1_ESM.docx]

Supplement 1. Overview of quantitative surveys among care providers regarding knowledge, practice, referral and barriers about the discussing of fertility and FP with cancer patients of childbearing age; 1) conducted/published in the past 10 years, 2) excluding qualitative studies and studies concerning pediatric care providers.

| **Study** | **Year of survey** | **Country** | **Study population** | **Type of care providers** | **Eligible participants** | **Final participants** | **Completion rate (%)_a_** | **Female (a) and male (b) patients** | **Knowledge of FP (%)** | **Discussing fertility and referral (%)_b_** | **Two most mentioned barriers (%)** |
| --- | --- | --- | --- | --- | --- | --- | --- | --- | --- | --- | --- |
| ***** *et al*. (current survey, 2021) | 2013-2014 | The Netherlands | Dutch Society for Medical Oncology | Medical oncologists | 392 | 120 | 30.6% | a, b | 47.5% sufficient knowledge  76% wants to improve knowledge | Discussing fertility: 68.3% often/always. Referral: 44.6% men, 28.9% women | Prognosis is poor 53%, unlikely patient will survive 43.1% |
| Vesali *et al.[49]* | 2015-2016 | Iran | Attendees congresses | Radiation oncologists and hematologists | 131 | 103 | 78.6% | a, b | Knowledge GnRH/sperm cryopreservation 2.77 and 2.64 | Information provision FP 13.6%.  Referral 71.8%. | N.A. |
| Post *et al.[38]* | 2016 | United States | Nationwide | Radiation Oncologists | N.A. | 352 | N.A. | b | N.A. | Fertility counselling always recommended by 52% | N.A. |
| Sallem  *et al.[44]* | 2012-2013 | France | Nationwide | Oncologists | N.A. | 102 | N.A. | a, b | 14% knowledgeable regarding FP. | Discussing fertility by 46%, referral 22% | Poor prognosis 54%, urgent treatment 51% |
| Melo *et al.[33]* | 2013-2015 | Portugal | Clinical institutions, Portuguese Oncology Society | Doctors who assist female cancer patients | N.A. | 111 | N.A. | a | N.A. | Fertility risks discussed 65.7%, preservation 59.3%. Referral 7.38%. | Time, knowledge. |
| Louwe *et al.[30]* | Unknown | The Netherlands | Seven of eight regions (85% of the country). | Hematologists, oncologists, radiotherapists, surgeons, gynecologists | 406 | 280 | 69% | a |           -  -              -  -    Knowledge treatment effect 55-68% chemotherapy 55%, radiation 68%. FP knowledge 13-36%. | 79% usually or always discuss fertility issues. | Lack of available reproductive specialists 11%, insufficient time 10% |
| Keim-Malpass *et al.[23]* | 2014 | United States | NCI-designated cancer centers | Nurses | 94 | 52 | 55.3% | a, b | dsds uncertainty and concern, alongside higher levels of depression (not specific to fertility: CES‐D) and cancer or fertility‐related trauma s  N.A. | Rarely/never discuss FP by 73.1%. Referral N.A. | Lack of available information, patient cannot afford FP |
| Chung *et al.[13]* | 2016 | China (Hong Kong) | Clinicians working in Hong Kong | Oncology, hematology, gynecology, pediatrics, surgery physicians | 467 | 167 | 36.5% | a, b | dfsdfdsafdafdafasdfdasweewrsfasdfdsadfasfasewrghyureiahlfdasgdhhdgdh a a, b, b a, b  45.6% reported familiarity with FP. | Percentage of discussing N.A. 68.3% never referred for FP. | No time before treatment 60.6%, risk of cancer recurrence 53.8% |
| Takeuchi *et al.[47]* | Unknown | Japan | Nationwide | Physicians involved with cancer patients | 412 | 180 | 43.7% | a, b | N.A. | Dscdcfdfdfsfdfddfdafdasfafd  Discussing fertility 42.7% sometimes, always. Referral N.A. | Insufficient knowledge |
| Shah *et al.[46]* | Unknown | United States | Members Society of Gynecologic Oncology | Gynecologic oncologists | 1087 | 152 | 14% | a | erer  N.A. | Assess fertility status 68% always. Referral easy according 64.9%. | Predictors: number of young women, cancer stage. |
| Rosenberg *et al.[40]* | 2015 | United States | Oncologists involved in endocrine studies. | Medical oncologists caring for patients with breast cancer | 301 | 93 | 31% | a | N.A. | Discussing fertility 98%, referral 97% | Cost/insurance 47%; patient does not want to discuss FP 27%. |
| Micaux *et al. [34]* | 2015 | Sweden | National | Physicians in oncology/hematology | 821 | 329 | 55% | a, b | High knowledge male risk fertility 59%, female risk fertility/ early menopause 65% | Discussing fertility: 74% female, 70% male; discussing FP 57% female, 63% male | Poor prognosis 78%, patient anxious or overwhelmed by having cancer 54% |
| Krouwel *et al. [26]* | 2012 | The Netherlands | Members Dutch Oncology Nursing Society | Oncology nurses | N.A. | 421 | N.A. | a, b | Sufficient knowledge of FP options: 31.1%. | Discussion of FP in more than half of the cases 42.6% | Lack of knowledge 25.2%, poor prognosis 16.4% |
| Ghazeeri *et al. [18]* | 2012-2013 | Lebanon | Lebanese Society Medical Oncology, practitioners two medical centers | Oncologists, clinical practitioners (i.e. students, doctors and nurses) | 81 oncologists, all practitioners | 53 oncologists, 88 practitioners | 65.4% for oncologists | a, b | 92.3% of oncologists estimated sperm cryopreservation should be done. | 73.1% of oncologists routinely discussed, 39.6% regularly refers to fertility specialist | N.A. |
| Buske *et al.[11]* | 2011-2012 | Germany | Oncologists | Oncologists | Unknown. | 120 | Unknown. | a, b | 49.6% knowledge sperm preservation, 37.3% well informed about FP measures. | Discussing possible fertility impairment by 65.8%, FP by 65.3%. Referral by 96.6% | Poor prognosis 62.7%, over 35 years 47% |
| Biglia *et al.[8]* | Not reported. | Italy | Breast surgeons, oncologists | Representatives of all Italians regions | Unknown. | 181 | Unknown. | a | N.A. | 91% discusses fertility, 60% referral | N.A. |
| Louwe *et al. [31]* | Not reported. | The Netherlands | 2 comprehensive cancer centers | Gynecologists, oncologists, surgeons, radiotherapists, hematologists | 206 | 96 | 46.6% | a | N.A. | 62% took action to protect the ovarian function | Poor prognosis or need for immediate therapy 62%, costs |
| Adams *et al*. [1] | Not reported. | United Kingdom | Members doctors.net.uk of GMC registered doctors. | Medical and clinical oncologists | 1488 | 100 | N.A. | a, b | Sperm preservation 64% sufficient; ovarian preservation 82% insufficient. | Providing informed consent 97%, 45% routinely referral fertility specialist | Perception FP poor success rates 81%, lack of knowledge 63% |
| Duffy *et al.[16]* | Not reported. | United States of America | National; sample American Medical Association. | Oncologists, hematologists, radiation oncologists, gynecologists | 1088 | 344 | 31.6% | a, b | ±50% moderate/high knowledge confidence in fertility/ FP options. | Not reported. | Not reported. |
| King *et al.[24]* | Not reported. | United Kingdom | Cancer institutes, networks, associations. | Nurses, nurse specialists, oncology/surgery residents, surgeons, oncologists. | N.A. | 306 | N.A. | a | Knowledge local preservation options variable. | Always discussed 48%, most of the times discussed 34%. | Age (77%), disease status (37.9%) |
| Arafa *et al*. [3] | Not reported. | Saudi Arabia | 3 regions; Eastern, Jeddah and Riyadh. | Medical, radiation and surgical oncologists. | 180 | 103 | 57.2% | a, b | Familiarity with ICSI 49.5%. | Routinely discussing cryopreservation 41.7%. Referral 19.5%. | Type of cancer (92%), age of patient (87%) |

_a_ Eligible participants/final participants (%)

_b_ Discussing of FP depending on how questions have been phrased.

*N.A.:* Not available
